# GSK-3 Activity Is Critical for the Orientation of the Cortical Microtubules and the Dorsoventral Axis Determination in Zebrafish Embryos

**DOI:** 10.1371/journal.pone.0036655

**Published:** 2012-05-04

**Authors:** Ming Shao, Yushuang Lin, Zhongzhen Liu, Ying Zhang, Lifeng Wang, Changbin Liu, Hongwei Zhang

**Affiliations:** Key Laboratory of Experimental Teratology of the Ministry of Education, Key Laboratory of Animal Cells and Developmental Biology of Shandong Province, Life Science College, Shandong University, Jinan, China; Medical College of Wisconsin, United States of America

## Abstract

The formation of dorsal-ventral (D–V) axis is the earliest event that breaks the radial symmetry and determines the bilateral body plan of a vertebrate embryo, however, the maternal control of this process is not fully understood. Here, we discovered a new dorsalizing window of acute lithium treatment, which covers only less than 10 minutes after fertilization. Lithium treatment in this window was not able to reverse the ventralized phenotype in *tokkeabi* (*tkk*) mutant embryos, and its dorsalizing activity on wild-type embryos was inhibited by nocodazole co-treatment. These evidences indicate that the underlying mechanism is independent of a direct activation of Wnt/β-catenin signaling, but depends on the upstream level of the microtubule mediated dorsal determinant transport. In order to identify the target of lithium in this newly discovered sensitive window, GSK-3 inhibitor IX as well as the IMPase inhibitor L690, 330 treatments were performed. We found that only GSK-3 inhibitor IX treatment mimicked the lithium treatment in the dorsalizing activity. Further study showed that the parallel pattern of cortical microtubules in the vegetal pole region and the directed migration of the *Wnt8a* mRNA were randomized by either lithium or GSK-3 inhibitor IX treatment. These results thus revealed an early and critical role of GSK-3 activity that regulates the orientation of the cortical microtubules and the directed transport of the dorsal determinants in zebrafish embryos.

## Introduction

Dorsal-ventral axis formation is one of the earliest and vital developmental processes that determine the bilateral body plan of all vertebrate embryos. The dorsal organizer plays an important role in this process, and the molecular mechanisms of its induction have been elucidated before [1]–[7]. However, the upstream maternal control of the dorsal-ventral axis determination is still poorly understood for the moment. In *Xenopus* and zebrafish, the dorsal-ventral axis is determined shortly after fertilization. In *Xenopus*, fertilization triggers a “cortical rotation”, during which the egg cortex rotates with respect to the sperm entry point. Some proteins together with small granules and organelles move from the vegetal pole region to the perspective dorsal side by polarizedly aligned parallel microtubule arrays [8]–[10]. Although cortical rotation was not observed in zebrafish embryos [11], parallel microtubule arrays are also present at the vegetal pole about 20 minutes after fertilization (mpf) [12], [13]. Depolymerizing this microtubule arrays by UV, cold or nocodazole treatment leads to absence of the dorsal organizer and a ventralized phenotype [12], [14]. Vegetal yolk ablation before the first cleavage efficiently causes severely ventralized phenotype [15], [16]. These studies strongly indicate that some “dorsal determinants” (DDs) exist in the vegetal pole region of the zebrafish zygote. This hypothesis was further evidenced in a recent study, which identified the maternal-supplied *Wnt8a* mRNA as one of these determinants [17]. *Wnt8a* transcripts initially located in the vegetal pole after fertilization and were asymmetrically transported to one side of the yolk cortex in a microtubule dependent manner during the first several cell divisions [17].

The DDs are believed to trigger the Wnt/β-catenin signaling and cause the stabilization of β-catenin in the perspective dorsal region. The accumulated cytosolic β-catenin was observed to enter dorsal cell nuclei at about 128-cell stage in zebrafish embryos [18], [19]. The *ichabod* mutant harbors a mutation significantly reducing the expression level and nuclear localization of zebrafish β-catenin 2, which leads to the loss of organizer gene expression and severely ventralized phenotype [20], [21]. This ventralized phenotype can also be achieved by overexpressing Tob1, which can bind β-catenin and prevent the formation of β-catenin/LEF1 complex [22]. Nuclear β-catenin is missing in ventralized embryos caused by blocking the transport of the DDs, like the case in the *tokkeabi* (*tkk*) mutant, and early nocodazole or cold treated embryos [12], [23]. Activating Wnt/β-catenin signaling by overexpressing its components like Wnt3, Wnt8, CA-β-catenin, GBP, Dishevelled, dn-Axin1 or dn-GSK3β results in expansion or ectopic formation of the dorsal organizer, and can rescue or reverse the ventralized phenotype in *tkk* mutant embryos [23]. These studies put Wnt/β-catenin downstream of the DDs transport. Although the DDs model was established on solid evidence, the regulation of the DDs transport still needs further study.

Lithium salt, known as an anti-psychotic drug, is widely used to control the pathology of the bipolar disorder. The most accepted targets of lithium ion are GSK-3 and the phosphatidylinositol monophosphatase (IMPase) [24], [25]. GSK-3 is a component in Wnt signaling, which is inhibited after the canonical Wnt activation. Lithium can noncompetitively inhibit GSK-3 activity, probably by competing with Mg^2+^ for binding site in this enzyme [26]–[28]. Owing to this, lithium treatment can mimic the Wnt/β-catenin signaling activation by dephosporylating and stabilizing β-catenin, the direct substrate of GSK-3. And this is widely accepted to interpret the reason why lithium treatment at late cleavage stage causes dorsalization of vertebrate embryos [28]. As GSK-3 participates other metabolic processes and signaling transductions like insulin/insulin-like growth factor signaling, neurotrophic factor signaling and the phosphorylation of microtubule associated proteins [24], it can also regulate many other processes independent of Wnt signaling. IMPase is a key enzyme mediating inositol recycling in the IP_3_-DAG-Ca^2+^ signaling. Inhibiting this enzyme by lithium causes inositol depletion and eventual shutdown of the IP_3_-DAG-Ca^2+^ signaling, which is believed as the main mechanism for lithium's pharmacological effects on bipolar disorder [25].

It has been reported that acute lithium treatment at late cleavage stage can cause dorsalization of the zebrafish embryo via activating Wnt/β-catenin signaling. Previous studies only observed one sensitive window of lithium treatment [29]. Here in this study, an earlier sensitive window of lithium treatment was discovered, and this sensitive window is limited in an extremely short period, and lasts for only less than 10 min after fertilization. Although the target of lithium treatment in this window is still GSK-3, the mechanism is completely different from the 32-cell-stage lithium treatment, and depends on microtubule assembly. Further study revealed that the parallel alignment of the vegetal microtubule arrays in response to fertilization and the polarized migration of *Wnt8a* transcripts were randomized by GSK-3 inhibitors. Thus our study revealed for the first time that Wnt/β-catenin independent GSK-3 activity is required to regulate the orientation of microtubule arrays and the dorsal determinants transport, and also provided new insight to the different phases of the maternal control during zebrafish dorsal-ventral axis formation.

## Results

### 1. Dorsalizing activity of acute lithium treatment exists in two separate windows

**Figure 1:**
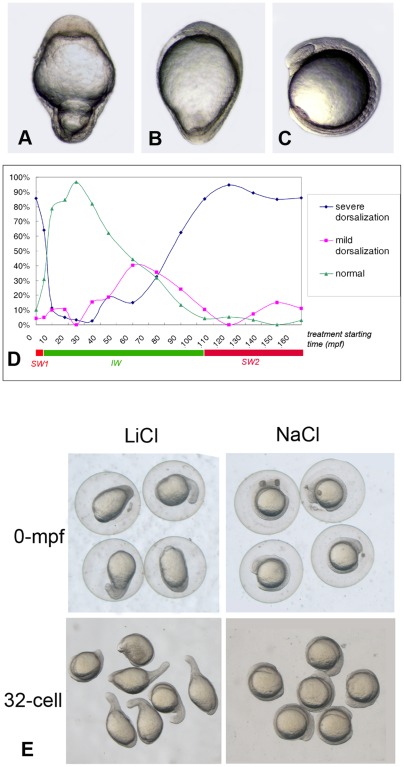
The dorsalizing activity of lithium treatment during zebrafish early development. (A) A severely dorsalized embryo (radialized). (B) A mildly dorsalized embryo. (C) A normal embryo. (D) Diagram demonstrating the dynamics of the dorsalizing capability of acute lithium treatment (0.3 M LiCl for 8 min). The abscissa axis designates the time at which lithium treatment began. The ordinate axis designates the percentage of three kinds of embryos with different degrees of dorsalization at 12.5 hpf. SW1: Sensitive Window 1; SW2: Sensitive Window 2; UW: Unresponsive Window. The data were obtained in three or more separate experiments, and the number of the embryos used for each data set is more than 100. (E) The dorsalizing effect of the lithium treatment is not caused by osmotic stress by comparing with NaCl treatment at the same salt concentration and treatment time. Embryos in A, B, C and E was at 12.5 hpf, and lateral viewed. The bar in A represents 500 μm.

Stachel et al. reported the dorsalizing activity of lithium treatment on zebrafish embryos and showed only one sensitive window from 32-cell stage to sphere stage, before which existed an unresponsive window with an earliest data obtained at 2-cell stage [29]. Here in this study, another sensitive window (SW1 in Figure 1D) was discovered, which was observed just after fertilization with a very short duration of about 10 minutes or less. The zebrafish embryos were synchronizely collected and were treated with 0.3 M lithium chloride (LiCl) solution for 8 min at specific developmental stages, and the phenotype was analyzed at 12.5 hours post-fertilization (hpf). The results showed that 85.6% of the embryos treated just after fertilization exhibited a radially dorsalized phenotype similar to the phenotype caused by 32-cell-stage lithium treatment [29]. These embryos were radially symmetric and showed a long elliptical shape at the end of gastrulation. The hypoblast cells streamed upwards from the circumference and accumulated at the animal pole (Figure 1A, C serves as a control). The percentage of these radially dorsalized embryos decreased significantly (lowered from 85.6% to 11.3%) when the lithium treatment was carried out 10 minutes later (Figure 1D). After the first cell division (45 mpf), zebrafish embryos gradually turned more and more sensitive to lithium treatment, with increasing percentage of partially dorsalized plus radially dorsalized embryos and decreasing percentage of the normal ones (Figure 1A, B, C, D). At the 32-cell stage (∼105 mpf), the dorsalizing activity of lithium treatment was comparable with that of 0 mpf lithium treatment (85.2% of radially dorsalized embryos, 11.4% of mild dorsalized embryos and 4.4% of normal). The dorsalizing activity of lithium lasts from 32-cell stage until the late blastula stage (Figure 1D), after which lithium treatment mainly caused anterior head truncation instead of dorsalization (data not shown). According to the curves in Figure 1D, we could define three different windows in zebrafish early development: sensitive window 1 (SW1, 0 mpf to 10 mpf), unresponsive window 1 (UW1, 10mpf to 32-cell stage), and sensitive window 2 (SW2, 32-cell stage to midblastula stage).

The osmotic stress of 0.3 M LiCl solution is about 53 fold higher than egg water. To exclude the possibility that any physical factor is responsible for the dorsalizing activity of lithium treatment, we used 0.3 M NaCl solution as control. The result showed that 8-minute treatment of 0.3 M NaCl at either 0-mpf or 32-cell stage had no effect on zebrafish embryogenesis, while most of the 0-mpf and 32-cell stage lithium treated embryos exhibited radially dorsalized phenotype (Figure 1E). These results indicate that the dorsalizing activity of lithium treatment in both windows is not dependent on physical factors like the osmotic stress, but on lithium-ion targeting biochemical processes.

### 2. Lithium treatment at 0 mpf causes the overall β-catenin nuclear localization and the expansion of organizer gene expression

**Figure 2:**
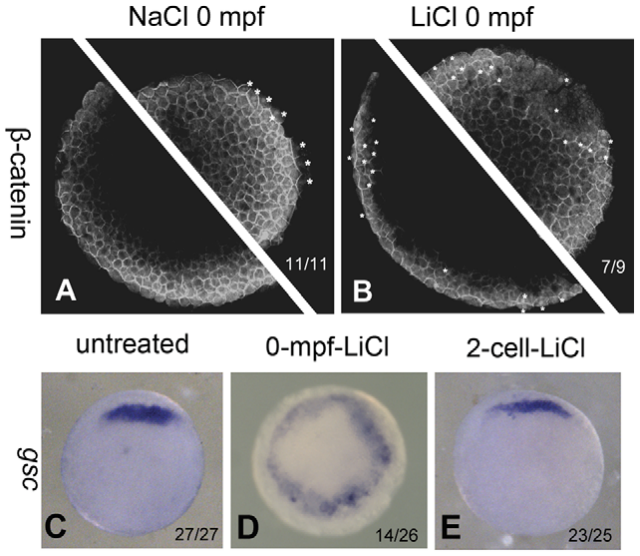
0-mpf lithium treatment activates Wnt/β-catenin signaling at mid-blastula stage and expands the organizer region. (A and B) Confocal immunofluoresence image of of β-catenin in a 0-mpf NaCl treated embryo (A) and a 0-mpf lithium treated embryo (B) To ensure that the entire marginal zone is investigated, each embryo was scanned for two focal planes near the marginal zone of two hemispheres. The embryos were at sphere stage. Nuclear β-catenin was marked with white asterisks. (C–E) Expression pattern of organizer gene *gsc* in untreated (C), 0-mpf lithium treated (D), and 2-cell-stage lithium treated (E) embryos. All the embryos were animal pole view, dorsal up if it can be distinguished.

The dorsal axis specification of zebrafish embryos is dependent on maternal Wnt signaling. Dorsal determinants (DDs) activate Wnt/β-catenin signaling in the prospective dorsal margin and stabilize the β-catenin protein. The stabilized β-catenin protein enters the nuclei of the dorsal yolk syncytial layer and the dorsal marginal cells and triggers the expression of downstream target genes like *bozozok*, *goosecoid*, *squint*, etc. [30]–[32]. Lithium treatment at 32–64 cell stage has been proved to enlarge the region where β-catenin enters the nuclei, and accordingly causes the expansion of dorsally expressed genes at the expense of ventral markers [19], [29]. To test if 0-mpf lithium treatment has such an effect, we stained the embryos at blastula stage using a β-catenin antibody. The result showed that the nuclear β-catenin appeared in the blastomeres located in all directions of mid-blastula embryos after 0-mpf lithium treatment (Figure 2B). In contrast, the nuclear β-catenin can only be observed in the dorsal marginal zone of NaCl treated embryos (Figure 2A). Next, we tested if the expression of the downstream organizer gene *goosecoid* (*gsc*) at 50% epiboly was altered by 0-mpf lithium treatment. We found that 0-mpf lithium treatment was able to expand the *gsc* expression region. More than half (53.8%, n = 26) of the embryos presented a circular expression pattern of this gene (Figure 2D), which is consistent with the radially dorsalized phenotype and the wide spread nuclear β-catenin. However, when lithium treatment was carried out at the 2 cell stage, no embryo presented the circular *gsc* expression and most (92.0%, n = 25) looked rather normal (Figure 2C and E). These results indicated that the 0-mpf lithium treatment can also cause widespread Wnt/β-catenin signaling activation in the blastula stage.

### 3. The 0-mpf lithium treatment perturbs a microtubule-mediated mechanism upstream of the Wnt/β-catenin signaling

It has been reported that the 32-cell-stage lithium treatment can directly inhibit GSK-3 and stabilize β-catenin, thus activating the canonical Wnt signaling, and this mechanism is responsible for the dorsalizing activity in the SW2 [28]. The lithium treatment in the SW1 can also cause the expansion of β-catenin nuclear localization and widespread organizer gene expression. Are the dorsalizing activities of lithium treatment in these two separate sensitive windows via the same mechanism, i.e. by directly activating Wnt/β-catenin pathway? To answer this question, we should first find a mutant strain with ventralized phenotype, and the mutant gene should function upstream of the maternal Wnt/β-catenin signaling. *Tkk* is such a maternal mutant in which the function of Kinesin binding protein Syntabulin is lost, so that the transport of the DDs from the vegetal pole to the perspective dorsal region was inhibited, and the embryo exhibits ventralized phenotype [13]. 32-cell-stage lithium treatment can rescue or even reverse the ventralized phenotype of the *tkk* embryos (personal communication from Dr. Hibi), so if 0-mpf lithium treatment functions via the same mechanism, the ventralized phenotype should also be reversed.

**Figure 3:**
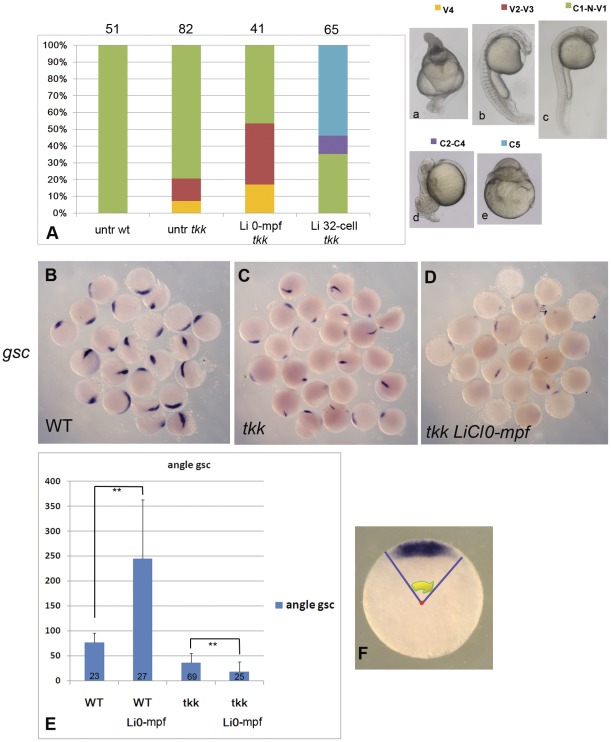
0-mpf lithium treatment exacerbates the ventralized phenotype of *tokkeabi* mutant embryos. (A) Phenotypic analysis of lithium treated *tkk* embryos. We adopted the Dorsoventral Index previously described [33], but some of the categories were combined in order to simplify the statistics, as stated below: (Aa) V4: a representative radially ventralized embryo; (Ab) V2-V3: a moderately ventralized embryo with distinguishable D–V axis but no eyes; (Ac) C1-Normal-V1: embryos with eyes (regardless of the size) and relatively normal D–V axis; (Ad) C2–C4: A partially dorsalized embryo with shortened anterioposterior length; (Ae) C5: A radially dorsalized embryo. (B–D) the expression of *gsc* in wild-type (B), *tkk* mutant (C), and 0-mpf lithium treated *tkk* mutant embryos (D). (E) The central angle of *gsc* expression showing a significant decrease in 0-mpf lithium treated embryos with respect to wild-type untreated, 0-mpf lithium treated wild-type and *tkk* untreated embryos. (F) The measurement of the central angle of *gsc* expression. The error bars in (E) designate the standard deviation of each data set. ** means that the p value is lower than 0.001 according to the Student's t test. Embryo numbers were designated for each column in (A) and (E).

To test this hypothesis, we carried out 0-mpf and 32-cell-stage lithium treatment on both *tkk* mutant embryos and wild type embryos. The results showed that 0-mpf lithium treatment cannot rescue or reverse the ventralized phenotype, but in sharp contrast, it synergistically aggravates the ventralized phenotype. *Tkk* females were crossed with young AB males, and this cross often generated embryos with low percentage of ventralization. To show the synergistic effect, we used several batches of such embryos with low penetrance from this cross. In these batches, only 7.3% of embryos presented severely ventralized phenotype (V4), and 13.4% with moderately ventralized phenotype (V2-V3, Figure 3A, the classification of phenotypes is according to Kishimoto et al. [33], with modifications). When these embryos were subjected to 0-mpf lithium treatment, the percentage of severe ventralized embryos rose to 17.1%, and the partial ventralized embryos increased to 36.6%. As expected, 32-cell-stage lithium treatment can reverse the ventralized phenotype of *tkk* embryos: no ventralized embryos were observed in this group and more than 64.6% showed dorsalized phenotype (C2 C5, Figure 3A). To further test this phenomenon, *tkk* or wild-type (WT) embryos were analysed by *in situ* hybridization at 50% epiboly stage using the probe of *gsc*. As expected, the *gsc* expression domain was reduced significantly in *tkk* embryos compared to the wild-type, with 12.0% (n = 25) no expression. But when 0-mpf lithium treatment was applied to the *tkk* mutant embryos, the expression of *gsc* was even much weaker than untreated *tkk* embryos and the *gsc* negative embryos rose significantly to 43.5% (n = 23) (Figure 3B, C, D). By measuring the central angle of the *gsc* expressing crescent, we found that for wild-type embryos, 0-mpf lithium treatment greatly increased the average central angle from 76.7° to 245.0°. But for *tkk* mutant embryos, the same lithium treatment caused a significant decrease in the angle (from 36.2° to 18.3°) (Figure 3E and F). These experiments demonstrated that Wnt/β-catenin signaling activation cannot explain the dorsalizing activity of 0-mpf lithium exposure. In addition, the results also indicate a possible connectedness between the mechanism of 0-mpf lithium treatment and Syntabulin associated processes.

### 4. Comparison between 0-mpf and 32-cell-stage lithium treatment on dorsal-ventral gene expression

**Figure 4:**
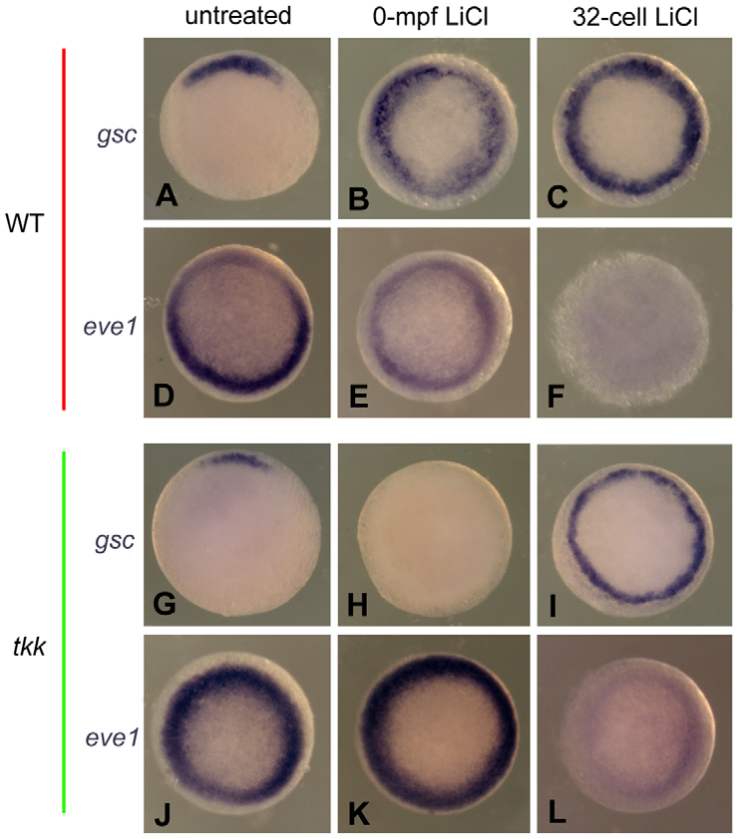
The comparison of dorsal and ventral gene expression between 0-mpf lithium treatment and 32-cell-stage lithium treatment. Representative embryos from indicated groups stained by *gsc* probe (A–C, and G–I) or *eve1* probe (D–F and J–L) at 50% epiboly. All the embryos are animal pole view and with dorsal side upward if it can be distinguished.

The dorsalizing activity of 0-mpf and 32-cell-stage lithium treatment is by way of different mechanisms, which may be reflected by differences in dorsal-ventral gene expression, although no phenotypic differences could be distinguished. To test this possibility, we reexamined by the *in situ* hybridization 0-mpf and 32-cell-stage lithium treated wild-type and *tkk* embryos at 50%-epiboly, using *gsc* and *eve1* as dorsal-ventral markers. As expected, several differences were discovered. 0-mpf lithium treatment usually caused a scattered expression of *gsc*, with distinct *gsc* negative cells in between, and the circular area of *gsc* expression was much thicker with respect to the untreated or 32-cell-stage lithium treated embryos (Figure 4A, B, C). *eve1* expression was reduced, but not absent in 0-mpf lithium treated embryos, while for 32-cell-stage lithium treated embryos, the expression of *eve1* frequently disappeared (Figure 4D, E, F). These results indicated that 0-mpf lithium treatment is less potent to induce dorsal gene expression or to inhibit ventral gene expression, which is in support of the possibility that 0-mpf lithium treatment altered the distribution of DDs rather than directly activated the Wnt/β-catenin signaling.

For *tkk* mutant embryos, 0-mpf lithium treatment caused a decrease or disappearance of the *gsc* expression and the enhancement of *eve1* expression (Figure 4G, H, J, K). But the 32-cell-stage lithium treatment can enlarge *gsc* expression region, and some embryos displayed a circular *gsc* expression and disappeared *eve1* expression (Figure 4I, L), although the frequency was much lower than the treated wild-type embryos. In addition, the width of *gsc* expression region was significantly thinner than the 32-cell-stage lithium treated wild-type embryos (Figure 4C, I), which is in accordance with the lower percentage of the radially dorsalized phenotype for 32-cell-stage lithium-treated *tkk* mutant embryos. These observations further support that the dorsalizing activities of the 0-mpf and the 32-cell-stage lithium treatment are via different mechanisms. They also suggest that *tkk* mutant embryos are less sensitive to the 32-cell-stage lithium treatment than the wild-type.

### 5. The dorsalizing activity of 0-mpf lithium treatment is dependent on microtubule assembly

**Figure 5:**
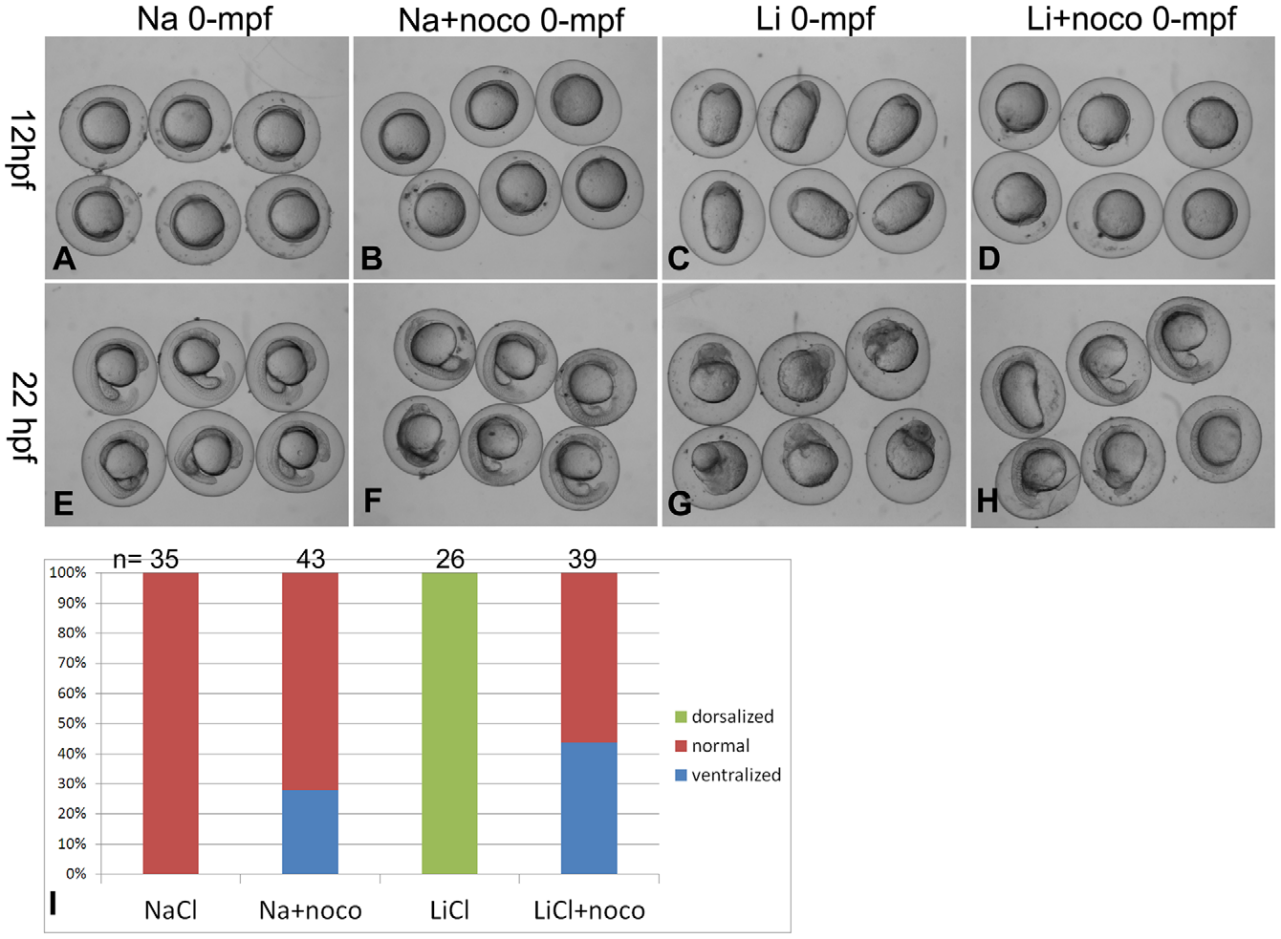
0-mpf nocodazole treatment reverses the dorsalizing effect of the 0-mpf lithium treatment. The 0-mpf embryos were treated with 0.35 M LiCl solution in the absence or presence of 0.1 μM nocodazole for 5 min, and then observed at 12 hpf and 22 hpf. 0 35 M NaCl treatment served as control. (A and E) 0-mpf NaCl treated embryos at 12 hpf (A) and 22 hpf (E). (B and F) 0-mpf NaCl and nocodazole co-treated embryos at 12 hpf (B) and 22 hpf (F). (C and G) 0-mpf lithium treated embryos at 12 hpf (C) and 22 hpf (G). (D and H) 0-mpf lithium and nocodazole co-treated embryos at 12 hpf (D) and 22 hpf (H). (I) Statistical data were obtained at 12 hpf for the experiment with embryo numbers on the top of each column.

Vegetal cortical microtubules align parallelly for the directed DDs transport after fertilization. Disrupting the microtubule assembly by nocodazole treatment stopped the polarized migration of *Wnt8a* mRNA and caused ventralization of zebrafish embryos [12], [17]. To verify the relationship between 0-mpf lithium treatment and cortical microtubule assembly, we tested if nocodazole treatment can reverse the dorsalized phenotype caused by the 0-mpf lithium treatment. The results showed that compared to 0-mpf NaCl treated embryos (Figure 5A, E), all the 0-mpf lithium treated embryos (26/26) showed a typical dorsalized shape with a significantly elongated animal-vegetal axis at 12 hpf (Figure 5C). However, when 0.1 μM nocodazole was added to the 0.35 M LiCl solution and treated at 0-mpf, all the 12 hpf embryos showed a much round shape, 43.6% embryos (n = 39) with more cells accumulated near the blastopore (Figure 5D), which is similar to the typical ventralized phenotype caused by NaCl-nocodazole co-treatment (27.9% ventralized, n = 43) (Figure 5B). At 22hpf, the 0-mpf lithium treated embryos showed dorsalized phenotype (radially or with curved or trunked tail) (Figure 5G), while for the LiCl-nocodazole co-treated embryos, ventralized phenotype with no head and enlarged yolk extension dominated the group (Figure 5H), very similar to NaCl-nocodazole co-treated embryos (Figure 5F). The changes in the phenotype after adding nocodazole demonstrated that depolymerizing microtubules can block the dorsalizing effect of 0-mpf lithium treatment (statistics shown in Figure 5I), which strongly indicated that the dorsalizing activity of the 0-mpf lithium treatment requires successful assembly of vegetal cortical microtubules.

### 6. The dorsalizing activity of 0-mpf lithium treatment is not due to the stabilization of microtubules

**Figure 6:**
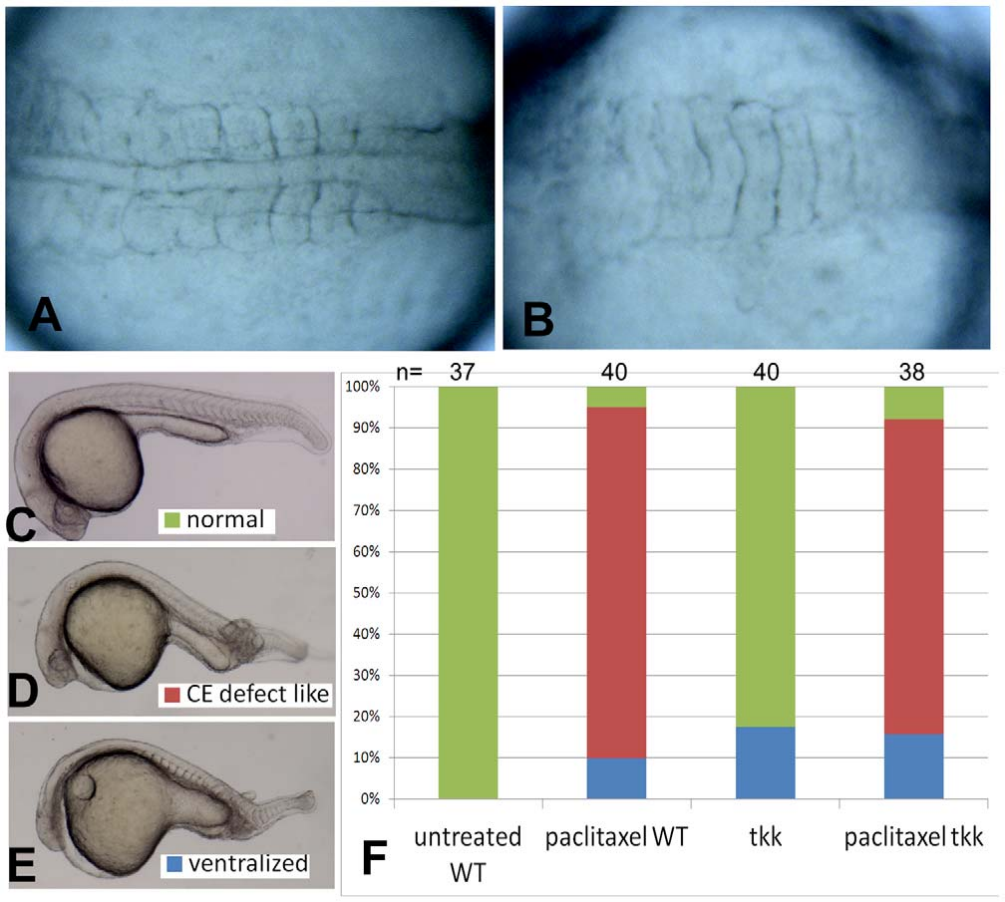
Paclitaxel treatment on wild-type and *tkk* mutant embryos. (A) Dorsal view of an untreated 12 hpf embryo. (B) Dorsal view of a 0-mpf paclitaxel treated embryo with ventralized phenotype. (C) An embryo with normal phenotype. (D) An embryo with CE defect like phenotype, showing a smaller head, shorter anterior-posterior axis and malformed somites. (E) A ventralized embryo. Embryos in (C-E) were observed at 24 hpf. The statistical data based on the 24 hpf observation were shown in (F) with embryo numbers shown on the top of each column.

*Xenopus* embryos can be dorsalized by D_2_O treatment in the first cell cycle [34], the mechanism of which is stabilizing the microtubules, resulting in the expanded distribution of DDs. In this study, we showed that depolymerizing the microtubules by nocodazole can reverse the dorsalized phenotype caused by 0-mpf lithium exposure, so a question arises as to whether lithium's effect on dorsal-ventral axis formation is a consequence of microtubule stabilization. Therefore we treated the wild-type embryos at 0-mpf with paclitaxel, a proved microtubule stabilizer. Interestingly, and unexpectedly, no dorsalized embryos were obtained after 0-mpf 7.5 μg/ml paclitaxel exposure, and on the contrary, 10.0% (n = 40) treated embryos showed a typical ventralized phenotype with no notochord observed at 12 hpf (Figure 6B, A as an untreated control). Other treated embryos exhibited a relatively normal dorsal-ventral axis but with mild defective convergence-extension, small head and malformed somites (Figure 6D, C as an untreated control, E is a ventralized embryo). We also tested the effect of paclitaxel exposure on *tkk* mutant embryos, and accordingly did not observe any rescuing effect of the ventralized phenotype (Figure 6F). These results suggested that stabilized microtubules are not sufficient to mediate the dorsalizing activity of 0-mpf lithium treatment.

### 7. The dorsalizing effect of 0-mpf lithium exposure is owing to the inhibition of GSK-3 activity

**Figure 7:**
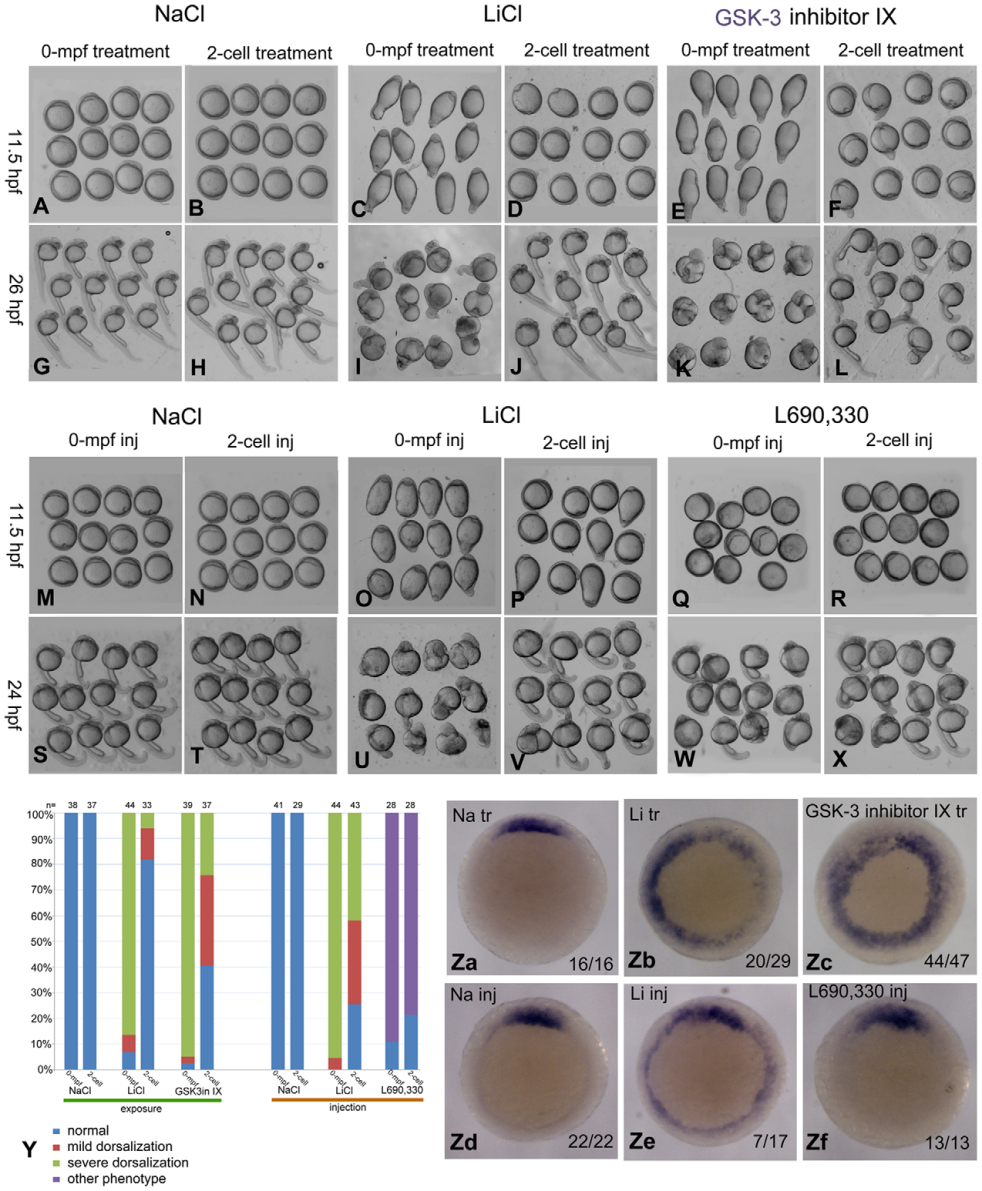
Comparison between lithium and chemical inhibitors of GSK-3 or IMPase in the dorsalizing activity. (A–L) Phenotypic comparison of NaCl, lithium and GSK-3 inhibitor IX exposures. (M–X) Phenotypic comparison of NaCl, lithium and L690, 330 injections. Embryos in A-X were observed at stages indicated at the left side of the figure. (Y) Statistical data of the phenotypic analysis at 11.5 hpf with embryo numbers on the top of each column. (Za–Zf) Examination of *gsc* expression in 0-mpf NaCl, LiCl, GSK-3 inhibitor IX and L690,330 exposed (Za–Zc) or injected embryos (Zd–Zf).

Lithium inhibits GSK-3 and IMPase, so another important question is to distinguish which enzyme serves as the real target. To address this question, we further used much more specific chemical inhibitors including GSK-3 inhibitor IX and IMPase inhibitor L690, 330, to analyze which one can mimic the lithium treatment in the dynamic dorsalizing activity. Just like lithium treatment, nearly all the 0-mpf GSK-3 inhibitor IX treated embryos exhibited a long elliptical shape at 11.5 hpf, and the large cell aggregation at the animal pole was also observed at 26 hpf. The dorsalizing activity of 2-cell-stage GSK-3 inhibitor IX treatment became much weaker than the 0-mpf treatment, which is also very similar to that of lithium exposure (Figure 7A, B, C, D, E, F, G, H, I, J, K, L, Y). We then injected L690, 330 or LiCl at 0 mpf zebrafish embryos and compared the resulting phenotypes. Injection of L690, 330 resulted in embryos with a typical convergence-extension (CE) defect rather than the dorsalized phenotype. These embryos appeared spherical at 11.5 hpf and showed distinct dorsal-ventral axis at 24 hpf, although the length of the dorsal axis was much shorter. In sharp contrast, 0-mpf LiCl injected embryos only exhibited dorsalized phenotype. When the injection was performed at 2-cell stage, the dorsalizing effect lithium was reduced, however we failed to find such a phenomenon in L690, 330 injection experiment (Figure 7M, N, O, P, Q, R, S, T, U, V, W, X, Y). To confirm the phenotypic data at the molecular level, we performed *in situ* hybridization experiment on 50% epiboly embryos using the probe of *gsc*. As expected, most of the 0-mpf GSK-3 inhibitor IX and 0-mpf lithium treated embryos showed radial or expanded expression of *gsc*. However, all of the L690, 330 treated embryos showed a relatively normal expression pattern (Figure 7Za–Zf). These data demonstrated that the 0-mpf treatment of GSK-3 inhibitor IX but not L690, 330 can mimic lithium in the dorsalizing activity, which strongly suggested that GSK-3 activity is required for the dorsal-ventral specification shortly after fertilization.

### 8. 0-mpf GSK-3 inhibition disrupts the parallel pattern of cortical microtubules and the polarized migration of *Wnt8a* transcripts

**Figure 8:**
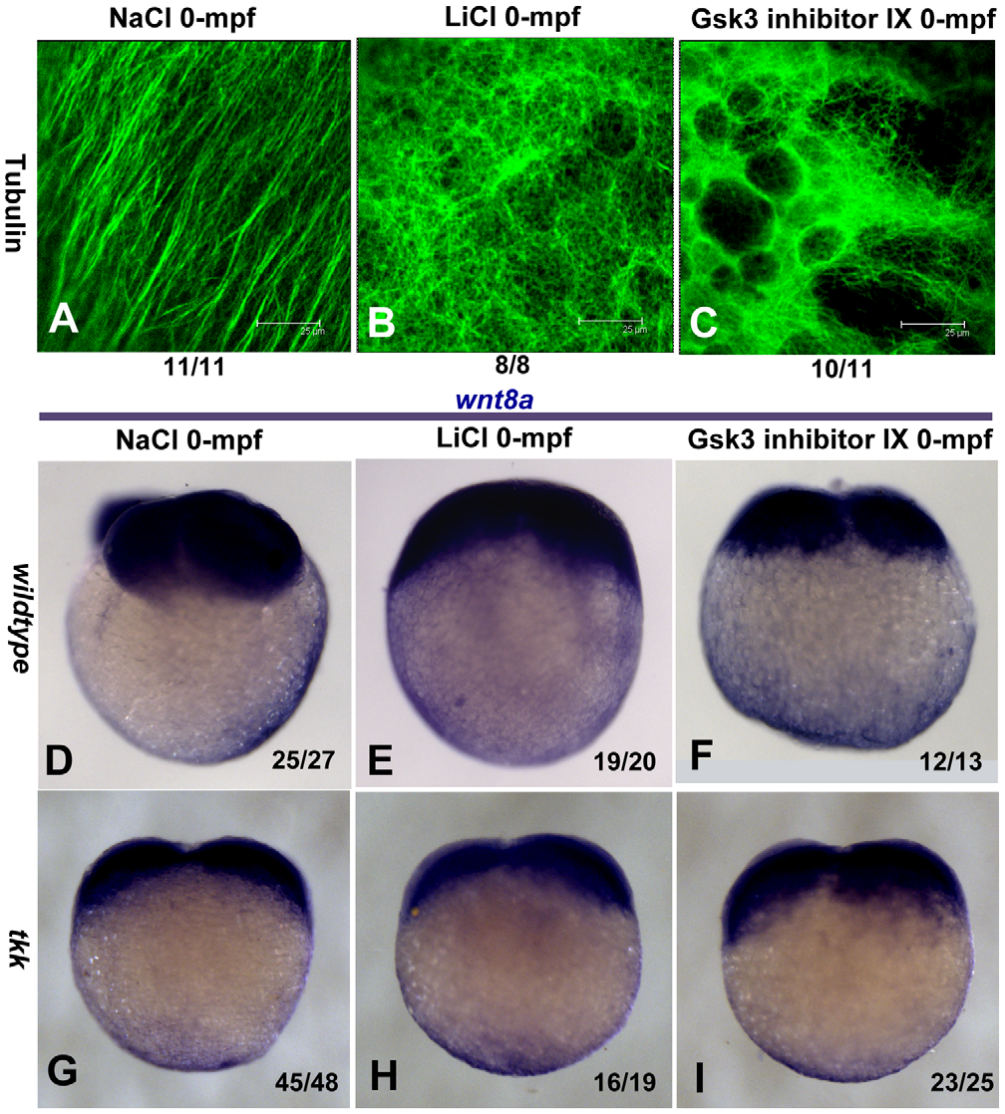
0-mpf inhibition of GSK-3 activity randomized the parallel microtubule arrays at the vegetal pole and the biased migration of *Wnt8a* transcripts. Microtubule staining with an anti-β-tubulin antibody to visualize the microtubule arrays formed at around 20 mpf at the vegetal pole. (A) Parallel microtubule arrays detected in NaCl treated embryos. (B) Randomized aligned microtubule arrays detected in 0-mpf lithium treated embryos. (C) Similar phenomenon detected in 0-mpf GSK-3 inhibitor treated embryos. (D–F) 0-mpf lithium and GSK-3 inhibitor IX treatments disrupted the polarized distribution of *Wnt8a* mRNA observed at 4-cell stage. (D) A 0-mpf NaCl treated wild-type embryo, (E) A 0-mpf lithium treated wild-type embryo, (F) A 0-mpf GSK-3 inhibitor IX treated wild-type embryo (G) *Wnt8a* mRNA restricted to the animal pole region of the 0-mpf NaCl treated 4-cell-stage *tkk* mutant embryos. (H I) Exposure of GSK-3 inhibitors failed to alter the distribution of *Wnt8a* mRNA in 4-cell-stage *tkk* mutant embryos.

It was reported that destroying the assembly of the microtubules by chemical or physical factors can ventralize vertebrate embryos [12], [14]. Blocking the microtubule dependent transport of DDs can also generate ventralized phenotype [21]. However, 0-mpf lithium treatment acts in an opposite way to generate the dorsalized phenotype, so it is impossible that 0-mpf lithium treatment can disrupt the assembly of cortical microtubules or inhibit the DDs transport. In fact, 0-mpf lithium treatment may affect the transport and broaden the distribution of DDs by influencing the remodeling of cortical microtubules after fertilization. To test this possibility, we stained and visualized the vegetal microtubule arrays formed ∼20 mpf by confocal microscopy. As reported, well-formed parallelly distributed microtubules in the vegetal pole region were detected in NaCl treated embryos (Figure 8A,), but in sharp contrast, the orientation of microtubules in 0-mpf lithium and GSK-3 inhibitor IX treated embryos were randomized and organized like an irregularly weaved net, and the microtubule bundles appeared much thinner with respect to control (Figure 8B, C). We further tested if the polarized migration of a recently identified DD, *Wnt8a* mRNA, was affected by GSK-3 inhibition. In 0-mpf NaCl treated 4-cell stage embryos, almost all the embryos (92.6%, n = 27) tested showed a biased distribution of *Wnt8a* in the yolk cortex (Figure 8D), but after inhibiting GSK-3 at 0-mpf by lithium or GSK-3 inhibitor IX, this asymmetric pattern disappeared, instead, the distribution of *Wnt8a* transcripts became much more smearing (Figure 8E and F). We also examined the expression of *Wnt8a* in 4-cell stage *tkk* mutant embryos, and found that in most cases (93.8%, n = 48), *Wnt8a* transcripts were restricted to the vegetal pole with indistinguishable biased distribution, and 0-mpf lithium treatment or GSK-3 inhibitor IX treatment did not alter their distribution in this mutant (Figure 8G, H, I), which was consistent with the previous data that 0-mpf lithium treatment failed to reverse the ventralized phenotype of *tkk* mutant. These observations suggest that GSK-3 activity is critical for the parallel alignment of the microtubule arrays after fertilization, and the dorsalizing activity of 0-mpf GSK-3 inhibition is probably the consequence of randomized microtubule arrays, which lead the DDs to a much broader area of the perspective marginal zone.

## Discussion

### 1. Lithium can dorsalize zebrafish embryos in two completely different ways

In this work, we have characterized another lithium-sensitive window to cause dorsalization of the zebrafish embryos. Lithium treatment carried out at the late cleavage stage was previously described for its capability to induce dorsalization in *Xenopus* as well as in zebrafish [29], [35]–[38]. However, the mechanisms of the dorsalizing effect of lithium treatment in the early (SW1) and late (SW2) sensitive windows are completely different. Lithium treatment at SW2 was reported to inhibit GSK-3, a negative regulator of Wnt/β-catenin pathway, thus can directly dephosphorylate and stabilize β-catenin, and activate the expression of the downstream dorsal organizer genes [28], [39]. 32-cell-stage lithium treatment can efficiently rescue or even reverse the ventralized phenotype caused by DDs deficiency in *Xenopus* and in zebrafish, indicating that the mechanism is downstream of the dorsal determinants (DDs) [36] (this study). If the dorsalizing activity of the 0-mpf lithium treatment functions through the same mechanism, these ventralized embryos should also be rescued. In fact, our data led us in an opposite conclusion that 0-mpf lithium treatment was unable to rescue the ventralized embryos by *tkk* mutation or nocodazole treatment (Fig 3, 4, 5). These results demonstrated that lithium treatment performed in SW1 and SW2 should impose their dorsalizing effect via different ways. This difference can further be sensed in two other phenomena: first, it seemed that zebrafish embryos in SW1 are more sensitive to lithium than in SW2. Slightly increasing treatment time (10 min e.g.) at 0-mpf often blocked or disrupted cell division and led to lethality before gastrula stage (data not shown), which is not the case for the 32-cell-stage lithium treatment. Second, the induction of dorsal organizer gene *gsc* by lithium treatment at SW1 or SW2 is different. This gene is normally expressed in an integrated manner in the dorsal marginal zones; 0-mpf lithium treatment often enlarged the expression region but made the positive cells discrete, while in contrast, 32-cell-stage lithium treatment caused a smoothly enlarged *gsc* expression region (Fig 4). This difference may be explained by the possibility that lithium directly activates Wnt/β-catenin pathway in the whole embryo in SW2, inducing *gsc* expression in all the marginal cells, but in SW1, lithium acts in a totally different way and the mosaic *gsc* expression is indirectly caused by a certain upstream mechanism.

**Figure 9:**
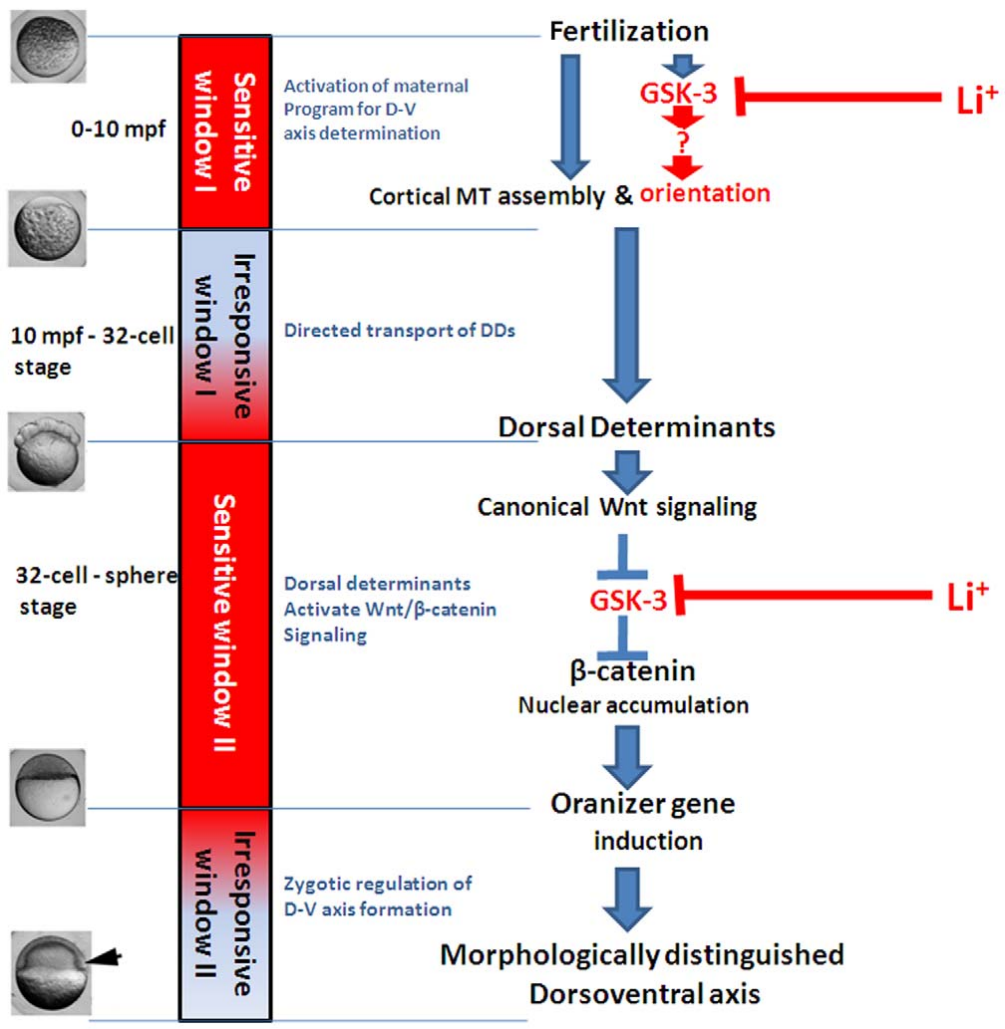
Model of dorsal-ventral axis formation in zebrafish. The zebrafish DV axis specification can be divided to four phases based on the dynamic alteration of the dorsalizing activity of lithium treatment. SW1 designates 0–10 mpf in which lithium treatment can cause dorsalization of the embryos. In this phase, fertilization initiates a GSK-3 dependent mechanism regulating the orientation but not stablization of vegetal microtubules which is critical for the dorsalward transport of DDs like *Wnt8a* mRNA. UW1 designates the first unresponsive window of lithium treatment, from 10 mpf to the 32-cell stage, in which, especially in the early period, lithium treatment fails to efficiently cause dorsalization. In this period, dorsal determinants are transported from the vegetal pole to the perspective dorsal side. The transduction of Wnt/β-catenin pathway is probably blocked by some unknown mechanism in SW1 and UW1. SW2 designates the period from the 32-cell stage to the mid-blastula stage. In this period, dorsally located DDs are able to inhibit GSK-3, causing the stabilization and nuclear localization of β-catenin, and lead to the expression of dorsal organizer genes. In UW2, lithium treatment loses its ability to dorsalize zebrafish embryos and the organizer gene expression is translated gradually by cell movement to morphologically distinguished dorsal-ventral axis. Arrow head at the lower-left corner indicates the shield.

The SW1 here is similar to the sensitive window of brief nocodazole treatment, in which the ventralized zebrafish embryos can be generated when nocodazole treatment is performed before 10 mpf, but the embryos were seldom ventralized when treated at 15 mpf [12]. As nocodazole treatment inhibits the microtubule polymerization, the similarity between SW1 and ventralizing window of nocodazole treatment may suggest that lithium also imposes its effect on the microtubule organization, but in a very different way. In this study, we presented three lines of evidence in support of this hypothesis. First, in the presence of nocodazole, the dorsalizing activity of the 0-mpf lithium treatment was completely lost (Figure 5), and the embryos only showed the ventralized phenotype, i.e. nocodazole treatment can override the lithium treatment, suggesting that the dorsalizing activity of 0-mpf lithium treatment requires the normal polymerization of microtubules. Second, 0-mpf lithium treatment was unable to dorsalize the maternal mutant *tokkaebi* (*tkk*) zebrafish embryos which harbors a mutation in the *syntabulin* gene encoding a protein required for the cargo transport along microtubule arrays [13]. Third, and most directly, we showed that the parallel microtubule array formed ∼20 mpf in the vegetal pole region was randomized by the 0-mpf lithium treatment, which raised a possibility that after 0-mpf lithium treatment, the transport of DDs is not directed to the perspective dorsal region but to the circumference of the margin, and this was further confirmed by marking a newly identified dorsal determinant, *Wnt8a* mRNA. These results thus put the mechanism of the 0-mpf lithium treatment at the upstream level to the microtubule dependent transport of DDs.

### 2. Wnt/β-catenin independent GSK-3 activity is required for the dorsal-ventral axis formation

GSK-3 and inositol monophosphatase (IMPase) are proved targets of lithium [25]. Our study using specific chemical inhibitors demonstrates GSK-3 as the real target of 0-mpf lithium treatment. GSK-3 plays an important role in Wnt/β-catenin signaling. However, our data indicate that directly activating Wnt/β-catenin pathway cannot be the cause of the dorsalizing activity of lithium treatment in SW1. In fact, GSK-3 inhibitors can randomize the alignment of vegetal cortical microtubule arrays and disturb the biased transport of the *Wnt8a* transcripts initiated by fertilization. Based on these observations, the dorsalizing effect of 0-mpf GSK-3 inhibition can be properly interpreted. In normal embryos, the active GSK-3 shortly after fertilization may facilitate the formation of parallelly aligned microtubule arrays, which is essential for the polarized transport of *Wnt8a* mRNA. But the polymerized microtubules failed to form parallel bundles after GSK-3 inhibition, instead, randomized and much thinner microtubule filament formed after lithium or GSK-3 inhibitor IX treatment. These net-like microtubule arrays can still transport DDs like *Wnt8a* transcripts, but might lead them to migrate in all the directions across the yolk cortex, so that many ventral lateral marginal cells receive sufficient dorsal-determining signals to change their fate. This deduction was supported by the fact that *gsc* positive cells often discretely distributed around the margin in 0-mpf lithium or GSK-3 inhibitor IX treated embryos. Syntabulin was thought as a linker between DDs and Kinesin motors during the microtubule dependent transport, so lacking this protein can reduce the amount of DDs transported to the dorsal margin (*Wnt8a* transcripts still located at the vegetal pole in 4-cell stage *tkk* embryos shown in Figure 8G), and causes ventralized phenotype. It can be imagined that the reduced DDs successfully transported in *tkk* embryos will be further diluted after their randomized migration caused by GSK-3 inhibition, and this dilution will make a sub-threshold supply of DDs for more marginal cells resulting in a more frequent appearance of ventralized embryos.

Data in this study also raised a question of whether GSK-3 regulates any microtubule-related protein independent of the Wnt/β-catenin signaling shortly after fertilization. It has been established that the microtubule associated protein Tau and MAP1B are substrate of GSK-3β (reviewed by [24]). Although no literature deals with the role that these proteins plays in the early D–V axis formation, it has been well studied that Tau is essential for microtubule stabilization in neuronal axons. Hyperphosphorylation and intracellular fibrillar formation of tau protein deter its ability to bind to and stabilize microtubules, and is a pathology found in Alzheimer's disease [40], [41]. Lithium is able to reduce the amount of phosphorylated Tau in cell culture [39], so it is conceivable that lithium may activate too much Tau by inhibiting the activity of GSK-3 after fertilization and randomize the vegetal paralleled microtubule arrays. This hypothesis, however, is far from being solidified. First, whether Tau owns a maternal expression is not verified. Second, our data showed that only stabilizing microtubules by paclitaxel treatment was not sufficient to dorsalize zebrafish embryos like GSK-3 inhibitors. Beside these doubts, another interesting aspect is that GSK-3 seemed to function oppositely in *tkk* mutant embryos for the unexpected ventralizing effect of lithium treatment. This phenomenon might indicate that the GSK-3 regulated process is extremely sensitive to slight disturbance of the cargo transport system, and the Syntabulin protein might functionally interact with GSK-3 or its substrate. Thus, further investigation is required to identify GSK-3 substrates responsible for the short-lived microtubule remodeling process. Functional analysis of these unknown molecules might shed light on the mystery of the instantaneous SW1 and the anomalous behavior of lithium treatment on *tkk* embryos.

### 3. The unresponsive window (UW) of lithium treatment during the early cleavage stage

It is very interesting that lithium had almost no effect on zebrafish embryos when treatment was performed during the first 1–2 cell cycles [29] (this study). Wnt signaling is not activated until midblastula stage, as revealed by TopdGFP transgenic zebrafish [42]. Our data also suggest that Wnt/β-catenin signaling cannot be efficiently activated by lithium treatment from fertilization to the late cleavage stage. There might be two possibilities for the existence of the unresponsive window: one is that the extra β-catenin stabilized by lithium treatment is degraded by an unknown negative feedback loop in the time span between the treatment and mid-blastula stage when Wnt signaling begins to activate, and the second possibility is that lithium treatment in this UW cannot rescue β-catenin from GSK-3. For the second possibility, we hypothesize that before the 32-cell stage, GSK-3 and its substrate β-catenin might be separated, and importantly at the same time, the average concentration of β-catenin is controlled below the activation threshold of the downstream cascade of Wnt signaling. GSK-3 is shown to be sequestered into the multi vesicular endosomes (MVB) in response to Wnt activation, and this phenomenon is required for the secondary axis induction in *Xenopus* embryos by Wnt ligands overexpression [43]. According to this, it is conceivable that GSK-3 and its substrate β-catenin might be segregated from each other by structures like MVB in the SW1 and UW, and GSK-3 is possible to be released gradually to the cytosol when cleavage continues under an unknown mechanism. According to this assumption, the existence of the unresponsive window can be properly interpreted: before the 32-cell stage, as very limited β-catenin can meet GSK-3, the lithium treatment in this stage can only increase a very small amount of β-catenin, which is not sufficient to activate organizer genes around the margin at mid-blastula stage. However, after 32-cell stage, as most β-catenin can contact the freed GSK-3 in the lateral-ventral regions, lithium treatment can stabilize large amount of β-catenin which may break the threshold to activate downstream target genes. To test these two models, more work is needed to verify the fluctuation of β-catenin before the 32-cell stage in control and lithium treated embryos, and to test if the maternal mutant with MVB formation defect is associated with the D-V axis formation problem in the zebrafish embryos, and if GSK-3 localizes in MVB before the late cleavage stage and is released to cytosol after the 32-cell stage.

### 4. Distinct phases of the dorsal-ventral axis specification revealed by lithium treatment, and remaining questions

Based on the data in this study, we could divide the D V axis formation process into four phases: 1) 0-mpf to 10-mpf, identical to the SW1, in this phase, fertilization induces a GSK-3 dependent mechanism that determines the orientation of vegetal microtubule arrays; 2) 10-mpf to 32-cell stage, corresponding to the UW1, in this phase, the dorsal determinants move directionally to the perspective dorsal marginal zone along paralleled microtubule arrays. 3) 32-cell stage to mid-blastula stage (SW2). Dorsal determinants begin to inhibit GSK-3 in the dorsal marginal zone, leading to β-catenin stabilization and nuclear localization, which further activate the early marker of the organizer; 4) Mid-blastula to early gastrula stage (UW2), in this phase, lithium treatment tends to induce posteriorization of neural system but not dorsalization. The dorsal-ventral axis specification is accomplished at the molecular level, and the asymmetric expression of dorsal-ventral genes is gradually translated to the morphologically distinct dorsal organizer structure–the shield (Figure 9).

So far, we know very little about what happens in the first two stages. Specifically, we know little about the signaling cascade that initiates and directs the transport of dorsal determinants; and we have not perceived the reason why GSK-3 inhibition cannot efficiently activate Wnt/β-catenin signaling in SW1 and UW1. More genetic and functional work is needed to identify and analyze maternal mutants with dorsal-ventral axis defect, which may shed light on these questions, and help to understand this earliest, vital, and intricate patterning process.

## Materials and Methods

### 1. Ethic statement

All embryos were handled according to relevant national and international guidelines. The study was approved by the Committee on the Ethics of Animal Experiments of Shandong University (Permit number: ECAESDUSM 2009035).

### 2. Fish Strains

Wild-type and the mutant *tokkeabi* (*tkk*) fish were used. The maternal mutant *tkk* embryos were generated by crossing wild-type male with *tkk* homogeneous female. The penetrance was evaluated by observing the phenotype at 24 hpf. The *tokkeabi* mutant strain is a gift from Dr. Hibi.

### 3. Egg collection, lithium, nocodazole, paclitaxel and GSK-3 inhibitor IX exposure

To ensure synchronic development of the zebrafish embryos, eggs were collected immediately when the female spawned. The fish mated in a chamber made up of three parts, an outer tank, an inner tank with narrow slits that allow the eggs to fall through to the outer tank, and a plastic sheet set in the middle of the inner tank to separate the male and female fish before mating. We kept a pair of fish separated in this chamber overnight and prepared an extra outer tank with egg water before the experiment. On the morning of the next day, the plastic sheet was removed and the fish began to mate. Once the female began to spawn eggs, the male and female were quickly separated by the plastic sheet and the inner tank was immediately transferred to the prepared extra outer tank. The synchronic eggs were collected immediately from the original outer tank and subjected to treatment or injection at indicated stages. Another batch of synchronic eggs can be collected by removing the plastic sheet again when convenient and repeating the procedure described above. In order to get sufficient synchronic eggs, the male and female fish were raised separately for more than a week before the experiment, and the female normally releases 20–40 eggs at one time during the mating. The data was obtained by several treatments with different batches of synchronic eggs. The collected embryos were treated with 0.3 M LiCl solution (diluted in E3 buffer) for 8 min at indicated stages. The nocodazole (Sigma, M1404-2MG) were dissolved in DMSO at a concentration of 5 mg/ml, and dilute in E3 buffer or mixed with the 0.35 M LiCl solution at a final concentration of 0.1 μg/ml. The 0-mpf treatment of LiCl, nocodazole or LiCl/nocodazole mixture was carried out immediately when the female fish spawned. As the embryos were extremely sensitive to nocodazole, so the treated time was reduced to 5 min, which is sufficient for 0.35 M LiCl to induce dorsalization. Paclitaxel (Sigma, T7191-5MG) was dissolved in DMSO at 1 mg/ml, then diluted to 7.5 μg/ml in an E3 buffer based solution containing 0.5 mg/ml Pronase (Roche, 11 459 643 001), and the embryos were treated in this solution for 8 min. GSK-3 inhibitor IX (Santa Cruz, sc-202634) was dissolved in DMSO at 10 mg/ml as a stock solution, and the embryos were exposed in a working solution of 10 μg/ml containing 0.5 mg/ml Pronase in E3 buffer for 8 min. The treated embryos were rinsed in E3 buffer for three times and incubated at 28.5°C until observation and fixation.

### 4. Microinjection

LiCl and NaCl were diluted in deionized water at a concentration of 0.15 M respectively, and was injected 2 nl to the 0–5 mpf and 2-cell-stage embryos. 2.5 mM L690, 330 (Santa Cruz, sc-202685A) aqueous solution was injected 2 nl for each embryo at the same stage to those used in LiCl and NaCl injections (the concentration of L690, 330 was used according to previous studies [44], [45]). The microinjection was performed using the MPPI-3 Pressure Injector.

### 5. Whole mount *in situ* hybridization

Sequences of *goosecoid* (*gsc*), *eve1* and *Wnt8a* were cloned in pGEM-Teasy vector. Anti-sense RNA probes were synthesized using the digoxigenin-UTP (DIG) in vitro transcription kit (Roche Applied Science, Indianapolis, IN, USA). Whole-mount *in situ* hybridization was conducted according to the zebrafish book [46].

### 6. Antibodies, immunofluorescence and confocal microscopy

The β-catenin localization was visualized by using an antibody from Abcam (ab6302), and the whole mount immunofluorecence was performed in a routine way as described [47], and the focal plane was selected near the margin; The microtubule was stained by an anti-β-tubulin antibody (Chemicon KMX-1), and the protocol were previously described [13], [48]. The focal planes were selected near the vegetal pole. The images were taken under a 10x objective of a Leica TCS SP2 confocal microscope.
