# Correction: GSK-3 Activity Is Critical for the Orientation of the Cortical Microtubules and the Dorsoventral Axis Determination in Zebrafish Embryos

**DOI:** 10.1371/annotation/e1bf2a26-2299-4126-bd5f-6710aacfb1e5

**Published:** 2012-06-05

**Authors:** Ming Shao, Yushuang Lin, Zhongzhen Liu, Ying Zhang, Lifeng Wang, Changbin Liu, Hongwei Zhang

There was an error in the published funding statement. The correct funding statement is:

This work is supported by China Postdoctoral Science Foundation (20100481275), Independent Innovation Foundation of Shandong University, IIFSDU (2010GN033, 2009TS082), the National Natural Science Foundation of China (31101038, 30570967, 30671072), and the 973 Major Science Programs (2007CB947100, 2007CB815800) from the Ministry of Science and Technology of China. 

